# Supplementary material for: Comparative Analysis of Transcriptome and Proteome Revealed the Common Metabolic Pathways Induced by Prevalent ESBL Plasmids in Escherichia coli
Source: Int J Mol Sci. 2023 Sep 12;24(18):14009. doi: 10.3390/ijms241814009 (PMC10531281; doi:10.3390/ijms241814009)
Supplement: Supplementary file 1 [file ijms-24-14009-s001.zip › ijms-2560745-supplementary.pdf]

Strains	*Doubling time*
J53	*33.99 ± 4.6*
J53/pCTXM123_C0996	*39.22 ± 1.9*
J53/pCTXM64_C0967	*41.84 ± 0.7*
J53/pHK01	*37.97 ± 3.3*
J53/pNDM-HK	*41.14 ± 2.9*
J53/pNDM-HN380	*39.00 ± 2.43*
J53/pJIE143	*49.08 ± 6.9*

Figure S1. Doubling times of J53 and J53 MDR plasmid transconjugants.

The growth of J53 and J53/pCTXM123_C0996, J53/pCTXM64_C0967, J53/pHK01, J53/pNDM-HK J53/pNDM-HN380, and J53/pJIE143 was measured in LB medium. The experiment was conducted in biological duplicates. Doubling times in the exponential phase were calculated.

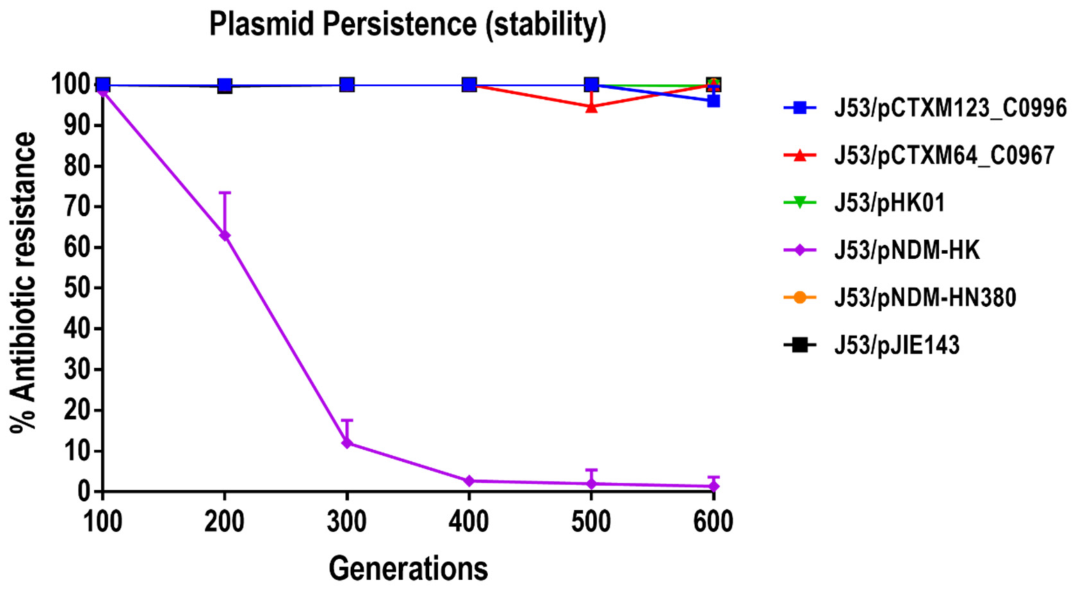

Figure S2. Plasmid persistence (stability)

The persistence (stability) of MDR plasmids was measured by the percentage of the remaining antibiotic resistance in MDR plasmid transconjugants cultured in antibiotic-free medium after specific generations. The antibiotic resistance of J53/pCTXM123_C0996, J53/pCTXM64_C0967, J53/pHK01, J53/pNDM-HK, J53/pNDM-HN380 and J53/pJIE143 were tested every 100 generations. The bars indicate the standard deviation. The experiment was conducted in biological triplicates.

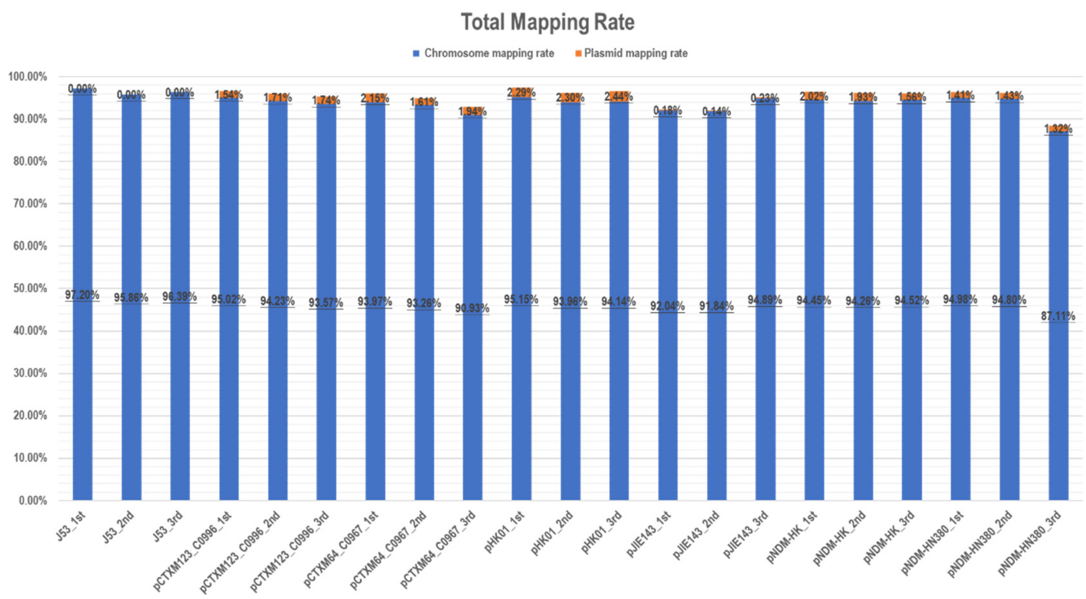

Figure S3. Mapping statistics of RNA-seq

The total mapping rate of RNA-seq at log phase is represented. Columns in blue represent sequencing reads that mapped to the chromosome of *E. coli* J53 in percentages. Columns in orange represent sequencing reads that mapped to MDR plasmids: pCTXM123_C0996, pCTXM64_C0967, pHK01, pNDM-HK, pNDM-HN380 and pJIE143, respectively, in percentages. The sum of the reads are the total mapping rates.

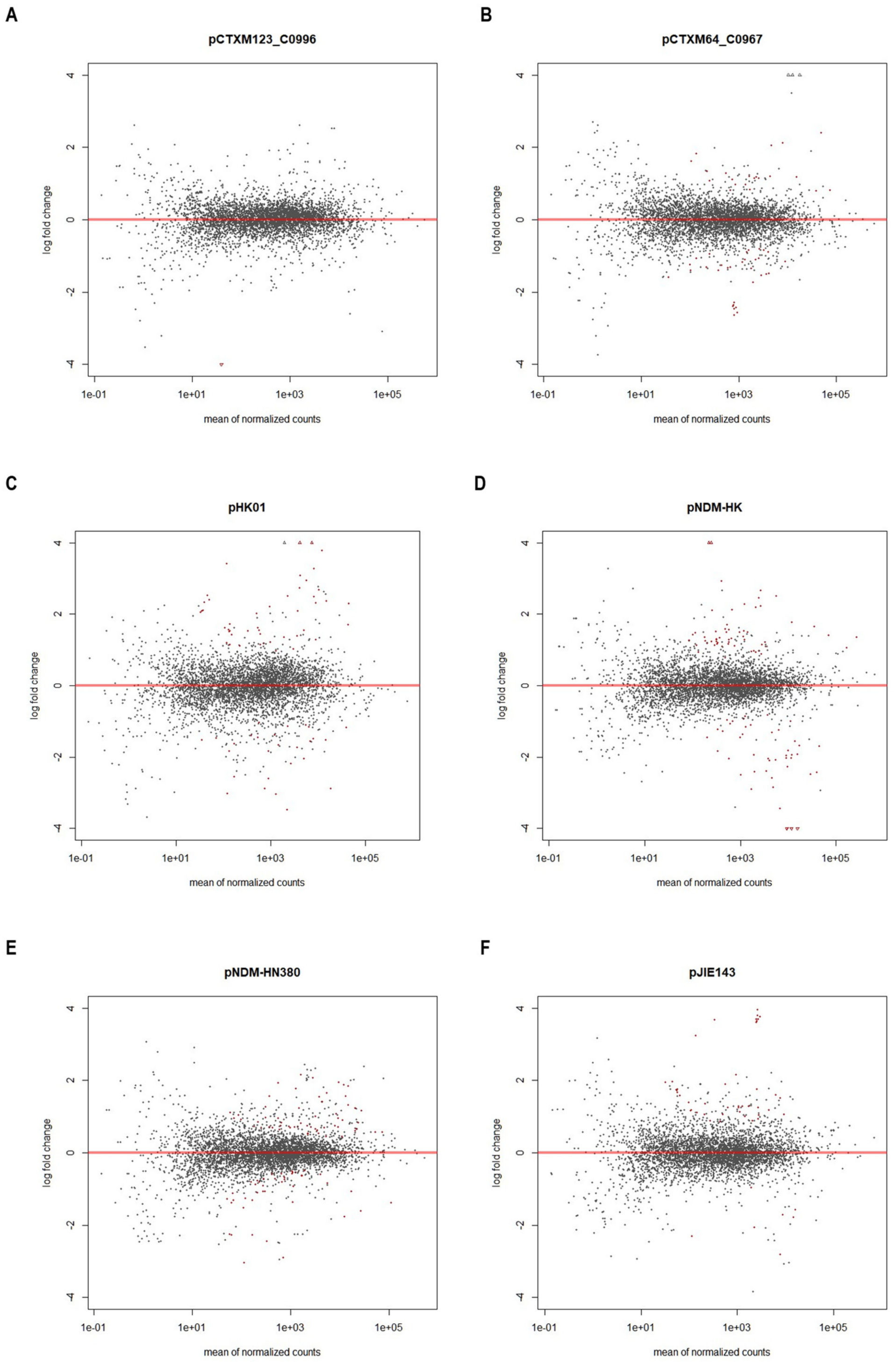

Figure S4. MA plot and DESeq2 analysis

(A-F) Mean of normalized counts vs. log2-fold change of genes of J53/pCTXM123_C0996, J53/pCTXM64_C0967, J53/pHK01, J53/ pNDM-HK, J53/pNDM-HN380 and J53/pJIE143, respectively. Each dot represents a gene. Dot is coloured in red if its adjusted p-value was less than 0.01.

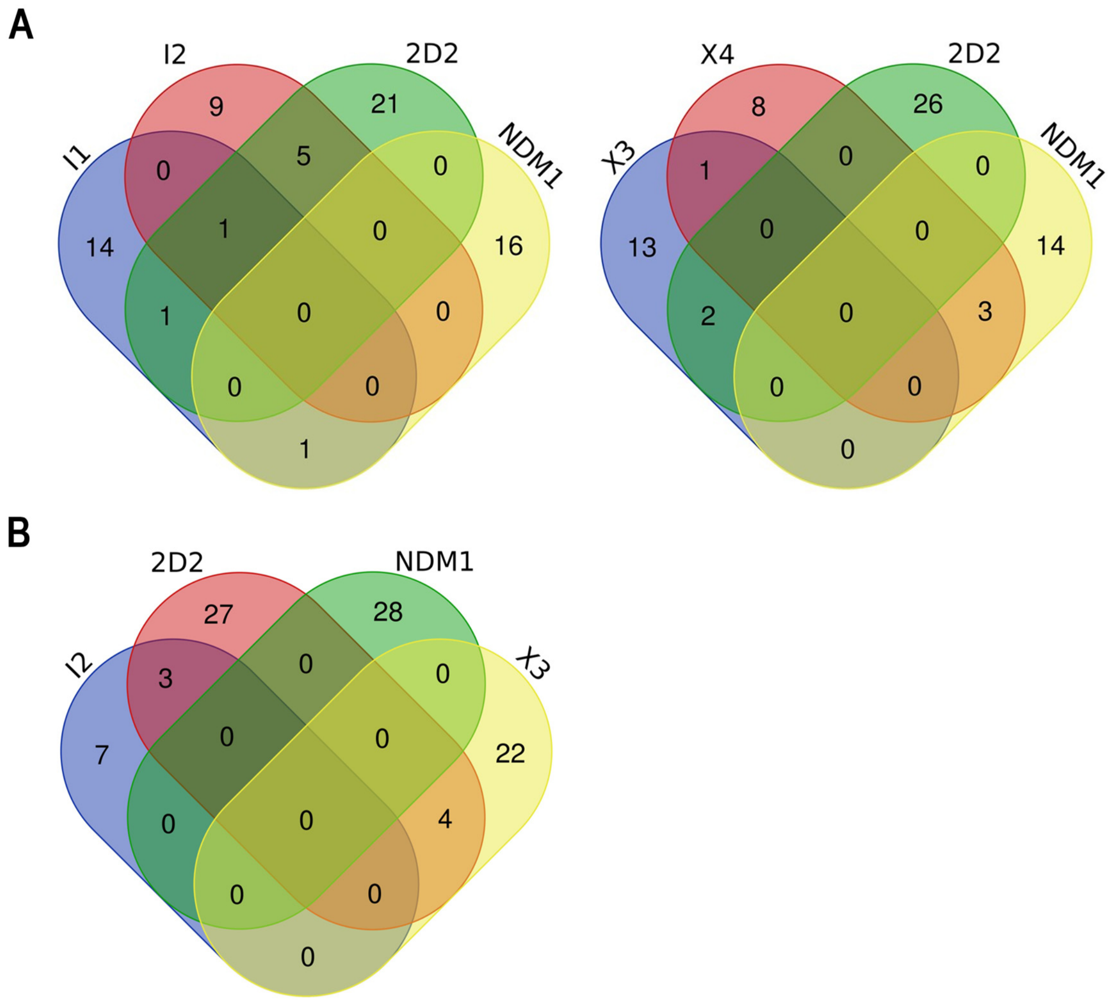

Figure S5. Differentially expressed genes in major MDR plasmids transconjugants

(A) Venn diagram of upregulated genes. Only genes with log2fold change >2 were included. (B) Venn diagram of downregulated genes. Only genes with log2fold change <-2 were included. I1: J53/pCTXM123_C0996, I2: J53/pCTXM64_C0967, 2D2: J53/pHK01, NDM1: J53/pNDM-HK, X3: J53/pNDM-HN380, X4: J53/pJIE143.

Workflow of Sample Level Enrichment Analysis (SLEA)

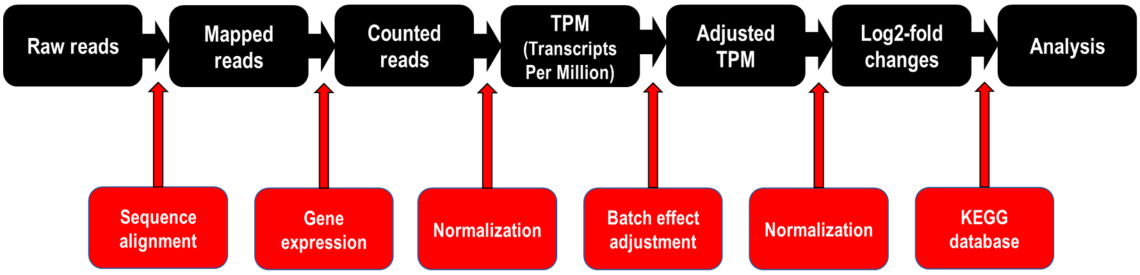

Figure S6. The work flow of Sample Level Enrichment Analysis (SLEA)

Raw sequencing reads were first aligned to the reference genome. The aligned (mapped) reads were than counted toward gene expressions. Read counts were than normalized in transcripts per million (TPM) and then adjusted to elimate the batch effect. The adjusted TPMs were than coverted into log2-fold changes to the controls. The log2-fold changes of genes were then imported to SLEA for analysis.

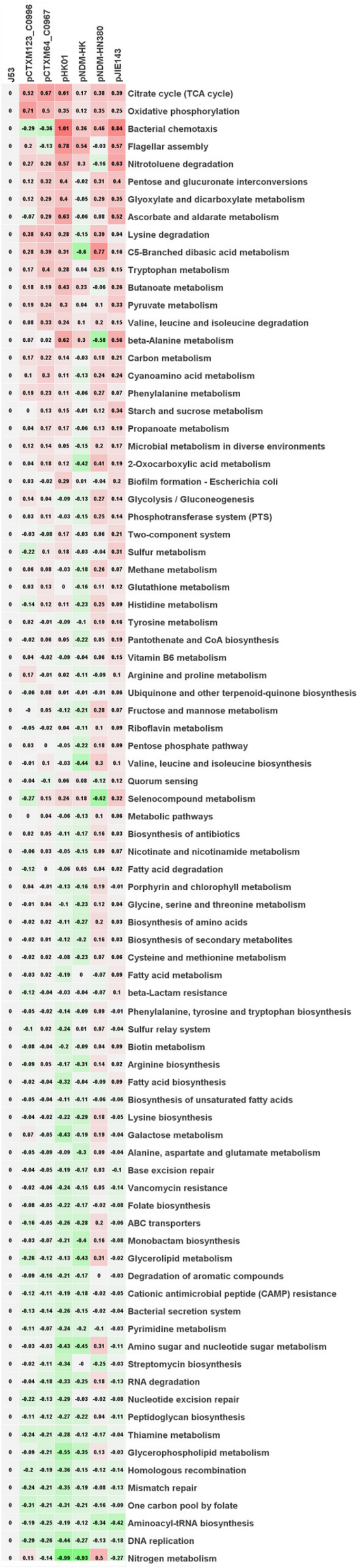

Figure S7. Heat map of SLEA at log phase

Overviews of KEGG pathway changes of MDR plasmid transconjugants. b-c. Top 10

upregulated and downregulated KEGG pathways of MDR plasmid transconjugants, respectively. The number in squares represents the log2-fold change of the pathways of the MDR plasmid transconjugant compared to J53. Upregulated pathways are coloured in red and downregulated pathway are coloured in green.

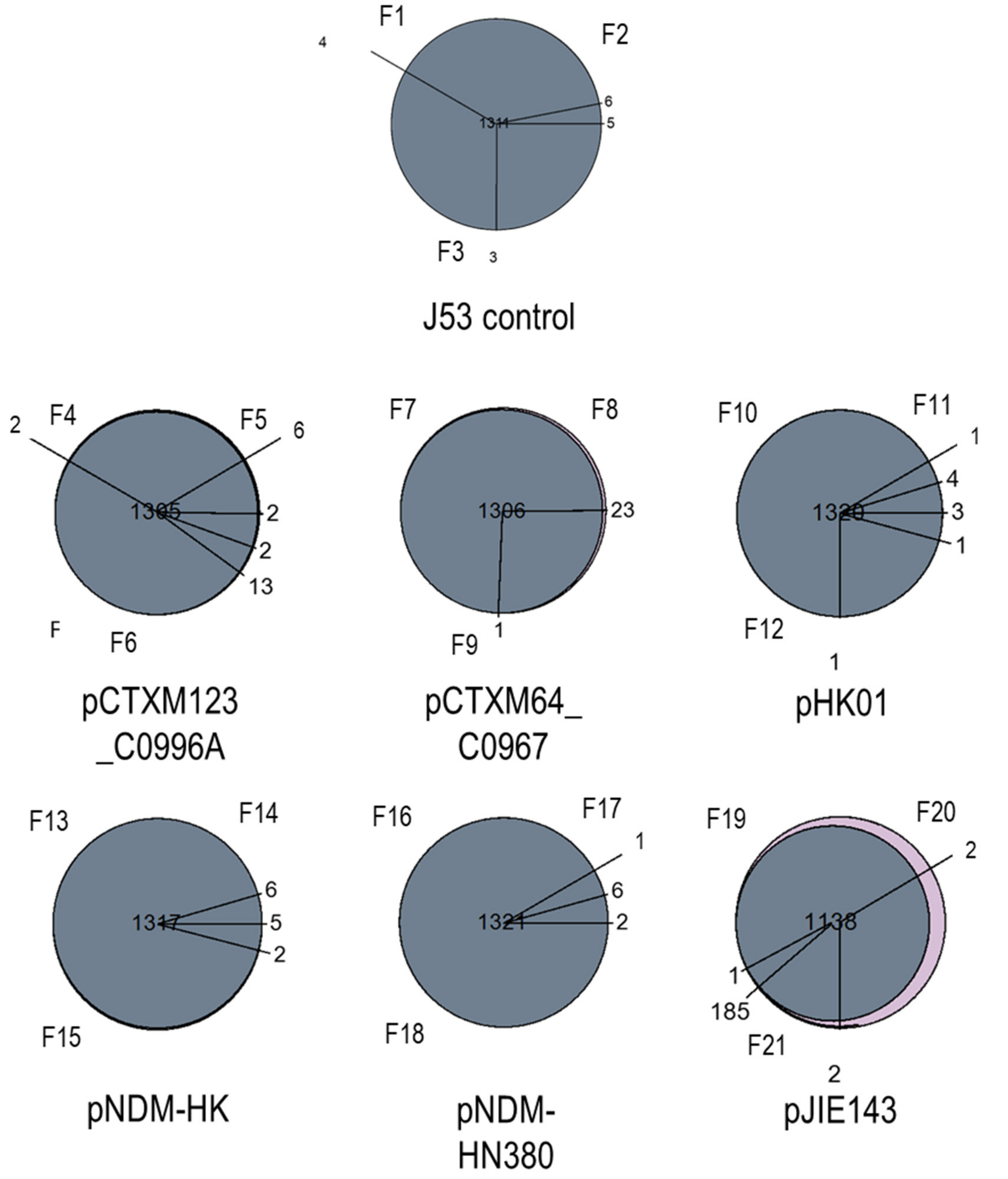

Figure S8. The overlap of proteins identified in each biological replication of six transconjugant harboring MDR plasmids and wild-type J53.

The replication of each strains are labeled as: J53 (F1-3), J53/ pCTXM123_C0996 (F4-6), J53/ pCTXM64_C0967 (F7-9), J53/pHK01 (F10-12), J53/ pNDM-HK (F13-15), J53/ pNDM-HN380 (F16-18), J53/ pJIE143

Table S1. Overview of RNA-seq samples

	J53	J53/ pCTXM123_C0996	J53/ pCTXM64_C0967	J53/ pHK01	J53/ pNDM-HK	J53/ pNDM-HN380	J53/ pJIE143	Total
Log phase	Yes	Yes	Yes	Yes	Yes	Yes	Yes	7

Table S2. Upregulated genes with log2fold change > 2

Plasmids	Total	Genes	Plasmids	Total	Genes
2D2 I1 I2	1	*dsdA*	I1	10	*ynfF yahV yobI ynfH ompL ynfG yfdN yjgZ cbeA phnF*
2D2 I1 X3	1	*dsdX*	I2	9	*ybcN rutC mdtN yddL pbl ytfA glpC yacM ais*
I1 NDM1 X4	1	*yhbO*	2D2	20	*nanM ydeQ nanT envR galT nanS nanE yhcH ariR nanA btsT mglA ythA yjhB mglC nanK nanC galE galK galM*
I1 X3	4	*ymjB rusA narK narG*	NDM1	14	*hycA hycD arrS ygeX yqeA hycC hydN xanQ ygfM racC xdhD ssnA ygeW ygeY*
2D2 I2	5	*galP glpD glpA glpT glpB*	X3	9	*lldD wcaF fepG ilvX nrdH narH nirB nrdI nirD*
2D2 X3	1	*nirC*	X4	8	*bdm ypjC yqfH yhaK ybdM tar lacY ydhY*
NDM1 X4	2	*hycB ynfP*			
X3 X4	1	*yfdT*			

I1: pCTXM123_C0996; I2: pCTXM64_C0967; 2D2: pHK01; NDM1: pNDM-HK; X3: pNDM-HN380; X4: pJIE143.

Table S3. Downregulated genes with log2fold change < -2

Plasmids	Total	Genes
2D2 I2	3	*bdm rusA ydaE*
2D2 X3	4	*kilR uacT racC ygeW*
I2	7	*ilvX yadW ymgK yhbO yqhI yfdM yfdT*
2D2	27	*ymjB hycA yqfG cheW yahV ibsA arrS pdeH yqeL fliD yfdN hycC cheR fliC tap yeeT motA adiC ycgR motB tar glcD ileY cheB ydhY cheA flxA*
NDM1	28	*nirC ychS hokA nanT dsdA glpF galP dicC nanS yhcH nanE glpD dsdX nirB btsT glpA yaiS glpT glpK yddA ythA yjhB glpQ glpC nanK nirD nanC glpB*
X3	22	*eaeH ygfK rcbA ygeK ygeX yfdS ynfH yehK ynfG ypdI yddY yecU hyuA xanQ ygfM ygbJ xdhD ssnA micF ygeY gspG ygeG*

I2: pCTXM64_C0967; 2D2: pHK01; NDM1: pNDM-HK; X4: pJIE143.

Table S4. List of plasmid-specific proteins identified in each transconjugant

	Accession	Description	Gene
**pCTX-M123_C0996 (pI1)**	H6V563	Beta-lactamase	*blaCTX-M-123*
	W0LWC0	Plasmid partition protein A	*parA*
	Q47396	Macrolide 2'-phosphotransferase	*mph(A)*
	D7UQM0	Fosfomycin resistance glutathione transferase	*fosA3*
	E9LLV2	Plasmid partition protein B	*parB*
	W0M036	FinO/Pro protein YafB	*yafB*
	A0A023UFL4	YedA protein	*orf00046*
**pCTX-M64_C0967 (pI2)**	C8CP57	Beta-lactamase	*blaCTX-M-64*
	M1EWW8	Uncharacterized protein	*M55_086*
	M1EXT8	Peptidyl-arginine deiminase	*yafB*
	Q9F576	CopG family transcriptional regulator	*yafA*
	M1EZQ8	YceB protein	*yceB*
	M1EWR9	FinO/ProQ protein YaeC	*yaeC*
	M1EVA3	Conjugal transfer protein	*TraL*
	Q9F552	Nickel transporter permease subunit C	*nikC*
	M1EWS7	Uncharacterized protein O	*M55_026*
	M1EXY1	Nickel transporter permease subunit B	*nikB*
	M1EXX7	Uncharacterized protein	*M55_035*
	M1EXU6	Uncharacterized protein	*M55_033*
	M1EXU1	Uncharacterized protein	*M55_018*
	M1EXZ9	Conjugal transfer protein	*M55_070*
**pHK01 (pFII)**	M1EXV7	RepA protein	*repA*
	Q9L5C7	Beta-lactamase	*blaCTX-M-14*
	E2GHG1	Maltoporin	*malB*
	E2GHH1	Plasmid partition protein A	*parA*
	Q6SIZ7	Conjugal transfer protein	*traT*
	Q6SIZ2	Alpha/beta fold family hydrolase	*yieA*
	E2GHG8	ABC transporter substrate-binding protein	*eitA*
	E2GHH0	Plasmid partition protein B	*parB*
	S5IKG2	Conjugal transfer protein	*virB6*
	E2GHM4	Conjugal transfer pilus assembly protein	*traC*
	Q6SJ23	Conjugal transfer pilus assembly protein	*traK*

	E2GHK0	Uncharacterized protein	*pHK01_051*
	E2GHI4	Uncharacterized protein	*pHK01_035*
**pNDM-HK (pL/M)**	E5KIY2	Beta-lactamase	*blaNDM-1*
	Q6SJ61	Beta-lactamase	*blaTEM-1*
	F1CNM7	Plasmid partition protein A	*parA*
	A7LI78	AAC(3) family aminoglycoside 3-N-acetyltransferase	*aacC2*
	F1CNQ3	Conjugation protein	*traH*
	F1CNQ0	Mobility protein C	*mobC*
	F1CNR5	Conjugation protein	*traR*
	F1CNR1	Conjugation protein TraN	*traN*
	F1CNM8	Plasmid partition protein B	*parB*
	F1CNK4	Uncharacterized protein	*D616_p59067*
	F1CNK7	mRNA interferase	*pemK*
	F1CNR3	Conjugative transfer protein	*traP*
	F1CNQ4	Conjugative transfer protein	*traI*
	F1CNK1	Thiol:disulfide interchange protein	*trbB*
	F1CNN1	Uncharacterized protein	*D616_p59081*
	F1CNJ9	Uncharacterized protein	*D616_p59068*
	F1CNN7	Uncharacterized protei	*D616_p59086*
	Q6F5A0	16S rRNA (Guanine(1405)-N(7))-methyltransferase	*armA*
	F1CNI6	N-(5'-phosphoribosyl)anthranilate isomerase	*trpF*
	F1CNM4	Uncharacterized protein	*D616_p59078*
	F1CNR7	Conjugative transfer protein	*traU*
**pNDM-HN380 (pX3)**	J7LFD4	60 kDa chaperonin	*groEL*
	A0A0D7L1W1	Beta-lactamase	*blaSHV-2*
	A3RID8	Beta-lactamase	*blaCTX-M-14*
	E9NWK5	Metallo beta lactamase	*blaNDM-1*
	G9G747	DNA-binding protein	*pCRE380_27*
	J7FS43	Bleomycin resistant gene	*bleMBL*
	M4Q855	Peptidyl-arginine deiminase	*AL524_RS00065*
	A0A0D7L1N6	HTH-type transcriptional regulator	*AB185_RS01320*
	J7LL31	IS5 transposase and trans-activator	*insH*
	A0A0K2CSL5	Uncharacterized protein	*p112298NDM_006*
	M4Q9L7	Yaja protein	*pCFNDM-CN_0050*
	J7LKG1	Transposase	*tnpA*
	M4Q5W6	DNA distortion protein 1	*taxA*
	A0A0F5LX25	IS110 family transposase	*AB182_RS02810*
	M4Q878	Incx type IV secretion system protein	*pilX6*

**pJIE413 (pX4)**	Q9EXV5	Beta-lactamase	*CTX-M-15*
	G8GYE3	Uncharacterized protein	*D616_p103009*
	G8GYG3	Acetyltransferase	*D616_p103033*
	G8GYE0	Peptidyl-arginine deiminase	*parA*
	G8GYG5	Conjugative transfer DNA nicking endonuclease	*D616_p103036*
	G8GYD9	Uncharacterized protein	*D616_p103003*
	G8GYF5	Inner membrane protein forms channel for type IV secretion of T-DNA complex,	*D616_p103023*
	G8GYG7	DNA repair protein	*D616_p103041*
	G8GYG0	Conjugal transfer protein	*D616_p103030*
	G8GYG9	Uncharacterized protein	*D616_p103044*
	G8GYE4	DNA-binding protein	*D616_p103011*

Table S5. List of DEP proteins identified in the mass spectrometry analysis

Gene name	UniProt Accession	Product	pI1	pI2	pFII	pL/M	pX3	pX4
narJ	P0AF26	molybdenum-cofactor-assembly chaperone subunit of nitrate reductase 1	1.51	1.38	1.53	1.33	1.57	1.46
tdcD	P11868	propionate kinase/acetate kinase C, anaerobic	1.33	1.45	1.46	1.25	1.86	1.61
tdcB	P0AGF6	L-threonine dehydratase, catabolic	1.16	1.29	1.22	1.03	1.71	1.45
dmsB	P18776	dimethyl sulfoxide reductase, anaerobic, subunit B	1.09	1.09	1.24	1.05	1.48	1.23
ychN	P0AB52	putative sulfur relay protein	1.09	0.82	1.50	1.06	1.17	1.19
garD	P39829	D-galactarate dehydrogenase	1.06	1.18	1.08	0.99	1.29	1.21
nrfA	P0ABK9	nitrite reductase, formate-dependent	1.05	1.18	1.31	0.26	1.42	1.20
tdcG	P42630	L-serine dehydratase 3, anaerobic	0.99	1.10	1.16	0.76	1.51	1.36
dmsA	P18775	dimethyl sulfoxide reductase, anaerobic, subunit A	0.99	0.82	1.04	0.78	1.23	0.96
tdcE	P42632	pyruvate formate-lyase 4/2-ketobutyrate formate-lyase	0.97	0.77	0.81	0.48	1.10	0.78
fdnG	P24183	formate dehydrogenase-N, alpha subunit, nitrate-inducible	0.90	0.62	0.29	0.13	1.13	0.10
glpB	P13033	anaerobic sn-glycerol-3-phosphate dehydrogenase membrane anchor subunit	0.74	0.71	0.82	0.62	0.81	0.69
glpC	P0A996	anaerobic sn-glycerol-3-phosphate dehydrogenase, C subunit, 4Fe-4S iron-sulfur cluster	0.69	0.65	0.74	0.56	0.80	0.74
narG	P09152	nitrate reductase 1, alpha subunit	0.68	0.76	0.78	0.60	0.79	0.82
nanT	P41036	sialic acid transporter	0.68	0.71	0.73	0.68	0.73	0.59
garL	P23522	alpha-dehydro-beta-deoxy-D-glucarate	0.67	0.59	0.75	0.67	0.73	0.67

		aldolase						
yjjI	P37342	DUF3029 family protein, putative glycine radical enzyme	0.67	0.69	0.73	0.48	0.92	0.78
garR	P0ABQ2	tartronate semialdehyde reductase	0.64	0.66	0.75	0.56	0.84	0.76
srlE	P56580	glucitol/sorbitol-specific enzyme IIB component of PTS	0.57	0.71	0.56	0.56	0.76	0.61
narH	P11349	nitrate reductase 1, beta (Fe-S) subunit	0.57	0.65	0.64	0.48	0.73	0.72
srlB	P05706	glucitol/sorbitol-specific enzyme IIA component of PTS	0.54	0.86	0.66	0.67	0.97	0.81
glpA	P0A9C0	anaerobic sn-glycerol-3-phosphate dehydrogenase, large FAD/NAD(P)-binding subunit	0.51	0.56	0.61	0.45	0.73	0.68
nanA	P0A6L4	N-acetylneuraminate lyase	0.47	0.55	0.60	0.57	0.64	0.51
ykgF	P77536	ferridoxin-like LutB family protein	0.46	0.53	0.42	0.24	0.76	0.62
tdcF	P0AGL2	putative reactive intermediate deaminase	0.44	0.50	0.44	0.26	0.82	0.55
ydeN	P77318	putative Ser-type periplasmic non-aryl sulfatase	0.44	0.58	0.56	0.33	0.74	0.71
srlD	P05707	sorbitol-6-phosphate dehydrogenase	0.44	0.71	0.54	0.59	0.82	0.52
pspC	P0AFN2	psp operon transcription co-activator	0.42	0.55	0.52	0.44	0.58	0.53
nirB	P08201	nitrite reductase, large subunit, NAD(P)H-binding	0.39	0.44	0.39	0.29	0.60	0.43
nanE	P0A761	putative N-acetylmannosamine-6-P epimerase	0.37	0.50	0.58	0.39	0.61	0.63
fepB	P0AEL6	ferrienterobactin ABC transporter periplasmic binding protein	0.34	0.83	1.32	1.08	0.86	1.07
ydfZ	P64463	selenoprotein, function unknown	0.26	0.33	0.35	0.41	0.70	0.36
cadA	P0A9H3	lysine decarboxylase, acid-inducible	0.26	0.29	0.43	0.26	0.63	0.22

ompC	P06996	outer membrane porin protein C	1.55	-0.11	0.70	0.64	0.82	0.70
ompF	P02931	outer membrane porin 1a (Ia;b;F)	1.21	-0.24	0.40	0.29	0.59	0.53
panD	P0A790	panD;	1.01	-0.39	0.54	0.20	0.48	0.50
rraB	P0AF90	Regulator of ribonuclease activity B	0.81	0.08	0.26	0.25	-0.96	-0.73
lacZ	P00722	beta-D-galactosidase	0.62	-0.74	0.87	0.15	0.79	0.72
allR	P0ACN4	glyoxylate-inducible transcriptional repressor of all and gcl operons	0.41	-0.86	-0.37	-0.09	-0.69	-0.50
rlmB	P63177	23S rRNA mG2251 2'-O-ribose methyltransferase, SAM-dependent	0.09	-0.59	0.10	-0.52	-0.40	-0.53
yjhU	P39356	putative DNA-binding transcriptional regulator	0.03	0.67	0.02	-0.04	0.05	-0.02
apaH	P05637	diadenosine tetraphosphatase	0.02	-0.76	-0.24	-0.67	-0.37	-0.20
recJ	P21893	ssDNA exonuclease, 5' --> 3'-specific	-0.04	-0.44	-0.91	-0.34	-0.62	-0.14
rpoE	P0AGB6	RNA polymerase sigma E factor	-0.08	-0.19	-0.12	0.51	1.21	0.05
nikA	P33590	nickel/heme ABC transporter periplasmic binding protein	-0.15	0.42	0.54	0.22	0.98	0.66
waaU	P27242	lipopolysaccharide core biosynthesis	-0.16	-0.12	-0.22	-0.17	-0.62	-0.27
livJ	P0AD96	branched-chain amino acid ABC transporter	-	-	-	-0.14	-	-

		periplasmic binding protein	0.17	0.05	0.10		1.04	0.42
flu	P39180	CP4-44 prophage; antigen 43 (Ag43) phase-variable biofilm formation autotransporter	-0.21	-0.73	0.03	-0.18	-0.08	-0.11
fbaB	P0A991	fructose-bisphosphate aldolase class I	-0.40	-0.45	-0.79	-0.49	-0.51	-0.47
osmC	P0C0L2	lipoyl-dependent Cys-based peroxidase, hydroperoxide resistance; salt-shock inducible membrane protein	-0.51	-0.32	-0.86	-0.44	-0.66	-0.54
aceB	P08997	malate synthase A	-0.57	-0.47	-0.78	-0.41	-0.52	-0.36
aceA	P0A9G6	isocitrate lyase	-0.58	-0.57	-0.47	-0.39	-0.59	-0.35
glnE	P30870	fused deadenylyltransferase/adenylyltransferase for glutamine synthetase	-0.63	-0.80	-0.57	-0.63	-0.44	-0.62
gsiB	P75797	glutathione ABC transporter periplasmic binding protein	-0.83	0.08	0.03	0.11	-0.03	0.08
tar	P07017	methyl-accepting chemotaxis protein II	-1.19	-1.60	-0.36	-0.80	-0.97	-0.05

The coral shaded rows indicate the differentially expressed proteins (DEPs) that are up-regulated in all six transconjugants. The blue shaded rows indicate the DEPs that are down-regulated in all six transconjugants.

Table S6. Functional Annotation of Genes

Gene Name	***Function***	***Relevance to Study***
DmsA, DmsB	*Subunits of the dimethyl sulfoxide (DMSO) reductase*	*Allows E. coli to grow anaerobically on DMSO as a respiratory oxidant.*
EitA	*Iron transporter*	*Involved in metal ion transport, essential for bacterial growth and metabolism.*
GarD/GarL	*Involved in catalyzing the dehydration of galactarate*	*Highlights specific metabolic pathways influenced by MDR plasmids.*
galP, galM, galT	*Galactose salvage pathway genes*	*Downregulated in most transconjugants, suggesting a potential metabolic shift influenced by MDR plasmids.*
glpABC, glpDD	*Anaerobic glycerol-3-phosphate dehydrogenase operon*	*Upregulated in J53/pCTXM123_C0996, indicating its role in virulence factor production and metabolism.*
IbsC	*Toxic peptide disrupting membrane integrity*	*Indicates the potential cytotoxic effects of MDR plasmids on bacterial cells.*
LsrF, LsrR, LsrG	*Involved in Autoinducer 2 degradation and biofilm formation*	*Highlights the influence of MDR plasmids on bacterial communication and biofilm formation.*
narG, narH, narK, nib, nirD	*Nitrate reductase genes*	*Downregulated in specific transconjugants, indicating potential changes in nitrogen metabolism.*
NikB, NikC	*Nickel permeases*	*Involved in metal ion transport, essential for bacterial growth and metabolism.*
repA, parA, parB, traL, traU, traC, virB, pemK	*Plasmid maintenance proteins*	*Essential for the stability, replication, and transfer of MDR plasmids.*
RpoS	*Stress-induced RNA polymerase sigma S factor*	*Emphasizes the role of MDR plasmids in bacterial stress response.*
sdhA, sdhB, sdhC, sdhD	*Key genes in the TCA cycle*	*Upregulated in most samples, suggesting a potential enhancement in energy production and metabolism.*
tdcD, tdcB, tdcG	*Anaerobic threonine/serine degradation pathway genes*	*Active in the absence of glucose, indicating potential metabolic shifts under anaerobic conditions.*
